# Isokinetic and functional shoulder outcomes after arthroscopic capsulolabral stabilization

**DOI:** 10.1007/s00402-021-04290-4

**Published:** 2021-12-29

**Authors:** Ewa Breborowicz, Przemyslaw Lubiatowski, Marta Jokiel, Maciej Breborowicz, Jakub Stefaniak, Adam Zygmunt, Marcin Wojtaszek, Piotr Kaczmarek, Leszek Romanowski

**Affiliations:** 1 Orthopaedics, Traumatology and Hand Surgery Department, Poznan University of Medical Sciences,, 28 Czerwca 1956 no 135/147, 61-545 Poznań, Poland; 2 Rehasport Clinic, Gorecka 30, 60-201 Poznań, Poland; 3 Physiotherapy Department, Poznan University of Medical Sciences, 28 Czerwca 1956 no 135/147, 61-545 Poznań, Poland

**Keywords:** Shoulder instability, Isokinetic evaluation, Capsulolabral repair, Shoulder biomechanics

## Abstract

**Introduction:**

Shoulder stability is secured by dynamic and static stabilizers. Rotator cuff is responsible for dynamic stabilization. In cases of shoulder instability their activity is disturbed. Capsulolabral repair restores mainly static stabilization. This surgery treatment technique of shoulder instability was first described by Bankart in 1923. His idea, with further modifications, is commonly used up to this day. Evaluation of muscle shoulder recovery after stabilization should be one of the important criteria to allow patient to return to sport and work. However, not much isokinetic assessment after capsulolabral repair was described. The aim of this study were the following: the comparative assessment of the shoulder rotatory strength in patients following arthroscopic capsulolabral repair of unilateral anterior traumatic instability and clinical assessment with comparison of pre and post-operative results.

**Material and methods:**

Forty-five patients, 14 women and 31 men, with an average follow-up of 4.4 years were tested bilaterally for internal and external rotation strength at four angular velocities. ASES and UCLA tests were collected before and after surgery.

**Results:**

The values of peak moment and muscle power parameters were slightly lower for an operated shoulder in comparison to a healthy shoulder for the external rotation. Total work parameter in external rotation was significantly lower for the operated shoulder in comparison to the non-operated side. The internal/external muscle group balance was lower for the operated shoulder in comparison to reference values in the women group. Furthermore, both ASES and UCLA scores were significantly higher after operation.

**Conclusions:**

After arthroscopic capsulolabral shoulder stabilization, slight differences in isokinetic evaluation, especially in external shoulder rotation, occur. It affects rotators muscle balance. In functional evaluation significant improvement in shoulder function occurs.

## Introduction

Shoulder stability is secured by dynamic and static stabilizers with the rotator muscles being responsible for the dynamic component. However, in cases of shoulder instability their work is disturbed [1, 2]. Capsulolabral repair restores mainly static stabilization, but the main purpose of postoperative rehabilitation was to rebuild dynamic stabilization. Thus evaluation of muscle shoulder recovery after surgical stabilization should be one of the important criteria for allowing patient to sport and work return.

Despite numerous reports describing capsulolabral repairs of the shoulder [3–5] very few assessed the muscular function. In their work, Amako et al. and Pavlik et al. studied the return of muscle strength after treatment of instability with open Bankart method [6, 7]. They suggested that return of full muscle strength was possible after 9–12 months. Amako et al. assessed results of arthroscopic stabilization as well indicating faster recovery of internal rotation (IR) compared to external rotation (ER) [2]. Tatha et al. described persistent loss of ER strength 12 months after arthroscopic stabilization [8]. Two other studies by Forthomme et al. and Felicetti et al. showed that there were no statistically significant differences between the operated and uninvolved RC muscles after 1 and 6 months following the Latarjet method [9, 10]. Rhee compared isometric muscle strength after open and arthroscopic shoulder stabilization reporting faster muscle recovery in arthroscopic group during first phase following surgery; 12 months later both groups displayed comparable results [11].

We have been using functional and proprioception testing routinely for evaluation of patients with shoulder instability [12, 13]. Based on our clinical observation of rehabilitation progress—usually our patients return to sport after 9 months. After literature review [2–11] we have hypothesized that the muscle parameters after 6 months after surgery should reach physiologic values. Therefore, the aim of this study was to perform isokinetic evaluation of the RC following arthroscopic capsulolabral repair of unilateral anterior traumatic instability. An additional goal was a clinical evaluation of shoulder function using subjective and objective assessment.

## Methods

### Patients

The study group comprised 45 patients, 14 women (average age: 37 y.o.; average weight: 71.4 kg; height: 170 cm; BMI: 24.6) and 31 men (average age: 30 y.o.; average weight: 82.5 kg height: 180 cm; BMI: 25.4) who had undergone arthroscopic labral repair in the Dept. of Orthopaedics, Traumatology and Hand Surgery, at Poznan University of Medical Sciences. In all patients metallic anchors were used. In those patients X ray evaluation did not show any signs of anchor pullout or loosening. After surgery patients were immobilized in Dessault-type shoulder brace for 4 weeks. After immobilization period patients begin rehabilitation. In the first phase they exercised with support to increase range of motion with slow gradual increase in external rotation. In the next phases patients performed strengthening exercises and proprioception training.

In our group there were six patients who were treated for recurrent instability. However, in all of them the evaluation according to chosen protocol was performed after reoperation. None of our patients has developed full instability after surgery—dislocations that have to be treated with reduction.

Patients were qualified based on the following inclusion criteria:

- unilateral traumatic anterior shoulder instability (TUBS)—type B2, according to Gerber’s classification (one direction instability without articular laxity, caused by trauma) [14],
- arthroscopic labral repair using suture anchors,
- no anchors loosening confirmed by radiological examination,
- minimal follow-up: 1 year, and
- no history of current or past contralateral shoulder problem.

Sixty consecutive patients that fulfilled the inclusion criteria were invited for the evaluation, out of whom 45 patients agreed to participate in study. The follow-up was 4.4 years (ranged from 1 to 12). Most of our patients were evaluated in the first 5 years after surgery (Table 1). We decided to analyze all patients together. We compared the results of patients in the most numerous groups and we have not found significant differences. None of our patient was attending any sports at professional or extensive level. All their activities were daily ones and leisure sport. We assumed this kind of intensity of activity would not increase the strength much when compared to longer and shorter period after surgery. All procedures performed were in accordance with the ethical standards of the institutional research committee (Ethical Committee at the Poznan University of Medical Sciences, resolution number 894/11) and with the 1964 Helsinki declaration and its later amendments or comparable ethical standards. All participants had signed written consent forms.

**Table 1:** The percentage distribution of years after surgery

Years after surgery	Number (*n*)	%
0 < *x* ≤ 2 years	13	28.9
2 < *x* ≤ 4 years	17	37.8
4 < *x* ≤ 6 years	4	8.9
6 < *x* ≤ 8 years	5	11.1
8 < *x* ≤ 10 years	3	6.7
10 < *x* ≤ 12 years	3	6.7

### Evaluation protocol

Evaluation protocol consisted of the following:

- Clinical evaluation according to ASES and UCLA scores. Results from pre and postoperative examinations were compared.
- Postoperative radiological evaluation (it was requirement for isokinetic test).
- Isokinetic examination at post-operative evaluation. Healthy and treated shoulders were compared.

Isokinetic shoulder testing was performed with a Biodex System 4 Pro® dynamometer. A bilateral protocol of IR and ER strength was based on testing at 180º/s (3 repetitions), 90º/s (3 repetitions), 360º/s (3 repetitions) and 270º/s (15 repetitions). Between each set there was a 15-s break for patient subjective recovery.

**Fig. 1:**
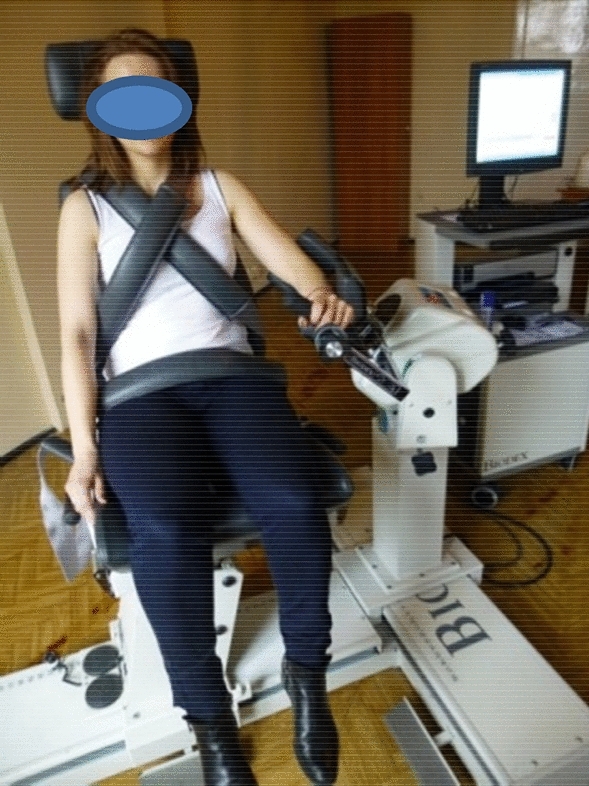
Patient position during examination

The patient was stabilized in the chair to eliminate any additional muscle compensation. The limb was stabilized in dynamometer forearm and arm supporter (Fig. 1). The shoulder was positioned in a safe position of 30°–45° abduction and testing was done in the scapular plane [15–17]. Patients’ warm up was provided by professional physiotherapists for each participant. There was verbal encouragement given by the examiner during the test.

We have focused on the highest value of peak moment (PM), the average value of peak moment/body weight (PM/BW), average power (AP), total work (TW) and the ER/IR ratio.

For clinical evaluation we used the ASES (American Shoulder and Elbow Surgeons) score, created by the Society of the American Shoulder and Elbow Surgeons. The ASES score contains a physician-rated and patient-rated section. Pain, instability and daily living activities’ assessment are included in the patient section. The physician-rated section contains range of motion, strength and instability evaluation as well as other clinical tests. The maximum number of points is 100 [18]. UCLA (University of California Los Angeles) score contains pain, strength, range of motion, function assessment as well as patient satisfaction. Points range from 2 to 35 in this scale [19].

### Statistical analysis

The statistical analysis was performed using Statistica®13 software (StatSoft). Power analysis and sample size were sufficient to provide valid results and comparisons. Normality tests were performed using Shapiro–Wilk test. Data with standard normal distribution were calculated with parametric *t*-Student test to assess the level of significance. For data with non normal distribution Wilcoxon signed rank test was used. To assess association between functional scales results and biomechanical parameters, the Spearman rank correlation with a conventional approach for interpreting correlation efficiency was applied. Moderate correlation was set 0.4 < *r* < 0.69 and values of 0.7 < *r* < 0.89 were set as strong correlation [20]. The level of significance was set at *p* < 0.05.

## Results

### PM, PM/BW, AP and TW

PM values in ER were slightly lower in the operated side when compared to the non-operated side in both man and woman group, in two velocities (270°/s in women group and 180°/ in men group); these differences were significant with *p* = 0.04 (Tables 2, 3).

**Table 2:** Values of peak moment (PM), peak moment/body weight (PM/BW), average power (AVG POWER), total work (TW) and average peak moment (AVG PM) parameters in external and internal rotation with comparison between operated and non-operated side in women group

Sex	Trial	External rotation										Internal rotation									
		PM		PM/BW		AVG POWER		TW		AVG PM		PM		PM/BW		AVG POWER		TW		AVG PM	
		nop	op	nop	op	nop	op	nop	op	nop	op	nop	op	nop	op	nop	op	nop	op	nop	op
WOMEN	90°∕s																				
	Avg	15.2	13.3	22.3	19.6	11.7	9.4	44.7	34.3	13.9	12.2	23.4	22.3	34.3	32.8	18.5	17.9	72.9	67.8	20.5	21
	SD	3.6	3.2	7.1	5.2	3.5	3.9	13.6	15.2	3.6	2.7	3.8	7.5	6.6	11.5	5.3	8.7	21.3	34.1	3.8	6.9
	p	NS		NS		NS		0.02*		NS		NS		NS		NS		NS		NS	
	180°∕s																				
	Avg	16	13.9	23.5	20.6	7.5	8.7	24	23.1	13.6	12	16.7	20.2	25	30	8.6	13.9	26.9	37.7	14	17.7
	SD	5.0	4.3	8.1	7.1	3.5	4.9	8.9	10.4	4.4	4.1	6.9	6.4	11.8	9.3	5.2	11.6	15.3	27.0	5.8	6.2
	p	NS		NS		NS		NS		NS		NS		NS		0.03**		0.04**		0.04**	
	270°∕s																				
	Avg	19.2	15.7	28.4	23.3	13.0	11.2	161.5	126.5	16.1	13.1	27.9	28.7	41.7	42.7	21.0	17.8	248.3	225.2	23.8	24.4
	SD	4.3	5.1	7.9	7.9	6.3	5.0	47.9	50.7	3.8	4.5	6.7	8.1	12.2	13.4	10.1	11.7	96.4	117.1	6.4	7.6
	p	0.04*		NS		NS		0.04*		NS		NS		NS		NS		NS		NS	
	360°∕s																				
	Avg	15.6	13.1	23	19.5	13.5	10.8	31.8	25.3	14.4	11.8	24.0	23.3	35.4	34.5	18.8	18.2	48.1	45.0	21.6	21.4
	SD	4.8	3.8	8.3	6.5	6.7	6.0	12.1	10.8	4.8	3.5	8.9	9.0	14.1	13.7	9.5	12.6	19.3	22.2	8.3	8.9
	p	NS		NS		NS		0.04*		NS		NS		NS		NS		NS		NS	

^*^t-Student test

**Wilcoxon test

**Table 3:** Values of peak moment (PM), peak moment/body weight (PM/BW), average power (AVG POWER), total work (TW) and average peak moment (AVG PM) parameters in external and internal rotation with comparison between operated and non-operated side in men group

Sex	Trial	External rotation										Internal rotation									
		PM		PM/BW		AVG POWER		TW		AVG PM		PM		PM/BW		AVG POWER		TW		AVG PM	
		nop	op	nop	op	nop	op	nop	op	nop	op	nop	op	nop	op	nop	op	nop	op	nop	op
MEN	90°∕s																				
	Avg	25.4	24.1	31.2	29.6	25.7	22.8	92.4	75.4	23.6	22.1	37.1	39.7	45.5	48.6	36.1	38.2	135.7	134.3	34.0	36.8
	SD	7.1	5.9	8.9	7.8	8.2	8.2	24.5	29.4	6.1	5.4	12.0	13.8	14.7	16.2	14.1	16.4	48.3	69.5	10.9	12.7
	p	NS		NS		0.005*		0.0003*		NS		NS		0.04*		NS		NS		0.01*	
	180°∕s																				
	Avg	24.3	22.9	29.9	28.2	22.0	23.0	53.2	49.7	20.7	20.2	30.2	33.2	37.0	40.9	30.1	40.4	74.5	88.9	24.6	30.6
	SD	7.6	7.2	8.9	8.9	13.0	12.9	23.3	25.2	6.6	6.6	14.3	12.2	17.5	15.3	23.8	26.8	47.5	50.8	11.6	11.4
	p	0.04*		NS		NS		NS		NS		0.01*		0.01*		0.0001*		0.005*		0.002*	
	270°∕s																				
	Avg	29.4	28.1	36.0	34.4	30.4	27.1	282.6	247.4	24.8	23.6	40.1	42.2	48.9	51.7	49.7	49.5	483.3	467.5	34.9	37.1
	SD	7.5	8.3	8.4	8.9	12.0	12.3	79.4	94.6	6.5	7.1	11.4	10.0	12.6	11.8	26.6	26.2	197.8	217.5	10.6	9.6
	p	NS		NS		NS		0.02*		NS		NS		NS		NS		NS		NS	
	360°∕s																				
	Avg	26.8	25.0	32.8	30.6	30.4	27.3	57.2	48.2	24.1	21.9	36.1	38.4	44.3	46.9	41.0	45.7	79.3	81.8	32.4	35.0
	SD	9.2	8.9	10.3	9.2	14.4	14.0	17.5	17.9	7.9	8.2	11.0	11.6	12.7	13.1	23.4	26.4	31.4	35.6	10.9	11.2
	p	NS		NS		NS		0.0004*		0.04*		NS		NS		NS		NS		NS	

^*^t-Student test

**Wilcoxon test

PM/BW and AP values were slightly lower in ER in operated shoulder compared to non-operated (Tables 2, 3).

The TW parameter proved to be significantly lower with p ranging from 0.04 to 0.0003 in ER for both women and men, in the operated shoulder compared to the uninvolved side, except velocity 180°/s in both groups (Tables 2, 3).

### The ER/IR ratio

Davies et al., Dvir et al., and Perrin et al. have determined in their work that the reference values of ER/IR ratio parameter should be in between 0.64 and 0.71 [21–23]. The external/internal rotation strength ratio values in operated shoulder in men group were 64% in 90°/s and 360°/s and 68% in 270°/s. These results corresponded with reference values. In women group, in operated shoulder the ratio was lower than reference values in all velocities except 180°/s. In both groups in 180°/s velocity ER/IR ratio was 73%, higher than reference values. In non-operated shoulder in both groups the ratio was almost always higher than reference results. Agonist/antagonist ratio results are summarized in Table 4.

**Table 4:**
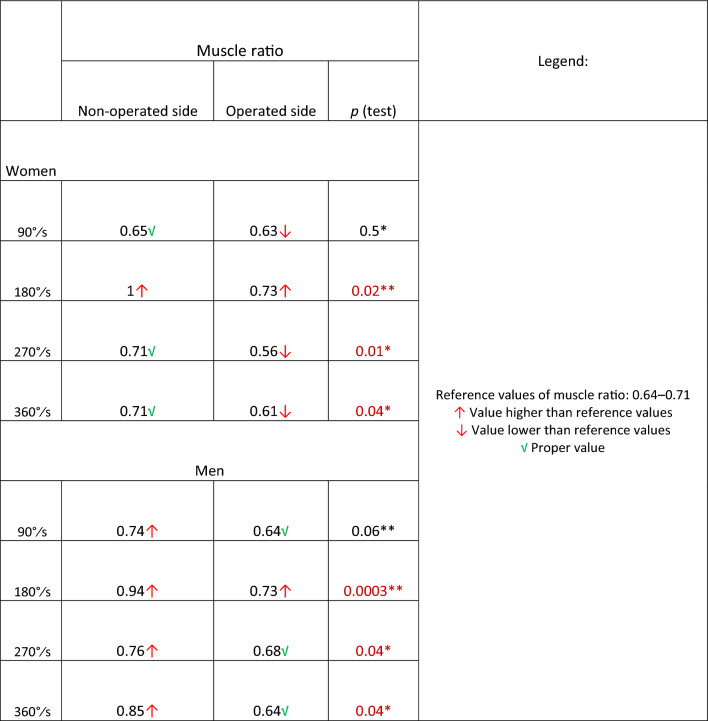
ER/IR ratio values with comparison between operated and non-operated side

^*^t-Student test

**Wilcoxon test

### ASES and UCLA scores

**Fig. 2:**
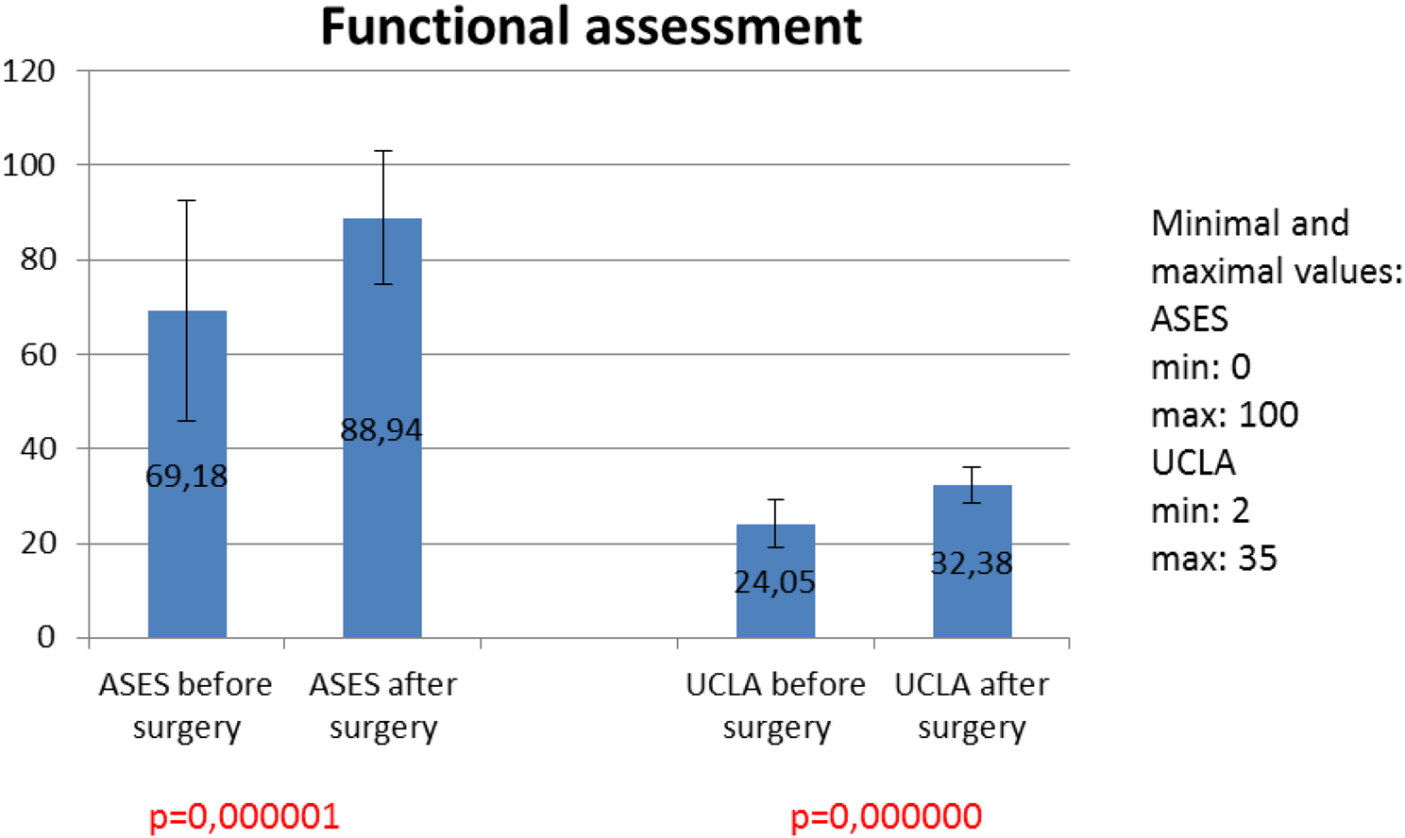
Results of clinical assessment mesured with ASES and UCLA scores before and after surgery

There was significant difference between ASES score before and after surgery with *p* = 0.000.001 (Fig. 2). Significant difference was also observed in UCLA scores before and after surgery with *p* < 0.000.001 (Fig. 2).

### Correlational analysis

This analysis revealed significant correlation between SSI score before surgery and some of measured biomechanical parameters in women and men group during involved limb external rotation (Table 5). There was no significant correlation between questionnaire scores and biomechanical parameters in internal rotation examination. In all examined groups there was a significant correlation between pain and UCLA score before and after surgery (Table 6).

**Table 5:** Correlation between ASES score before surgery and biomechanical parameters: peak moment (PM), peak moment/body weight (PM/BW) and average peak moment (AVG PM) in external rotation

External rotation	180°∕s	90°∕s	360°∕s	270°∕s
Women				
PM/BW				
*r*	0.33	0.62	0.65	0.53
*p*	NS	0.04	0.03	NS
MEN				
PM				
*r*	− 0.39	− 0.05	− 0.15	− 0.15
*p*	0.046	NS	NS	NS
PM				
*r*	− 0.39	− 0.11	− 0.09	− 0.13
*p*	0.05	NS	NS	NS

**Table 6:** Correlation between UCLA and pain before and after surgery in women and man group

	UCLA before	UCLA after
Women		
Pain before		
*r*	− 0.92	− 0.16
*p*	0.00001	NS
Pain after		
*r*	− 0.41	− 0.69
*p*	NS	0.02
Men		
Pain before		
*r*	− 0.44	− 0.61
*p*	0.02	0.001
Pain after		
*r*	− 0.08	− 0.72
*p*	NS	0.00001

## Discussion

The appropriate strength balance of shoulder muscles is extremely important for a shoulder’s proper function and is one of the most important factors in prevention of further injuries or recurrence of shoulder instability [24]. Thus, recovery of muscle strength and its related parameters seems to be crucial to regain optimal shoulder function after instability period which was followed by the labral repair. Isokinetic evaluation allows to assess a shoulder strenght. The changes can be correlated to the shoulder function. Although the PM is the classical strength outcome parameter [21, 22, 25, 26], for the evaluation of muscle endurance, total work may be equally or even more suitable [22, 25]. To obtain the most reliable result, the test should be measured at a speed of 90–450°/s with no more than 50 repetitions [22, 26–28]. According to literature review the values should be measured at a speed of 90–450°/s with no more than 50 repetitions. It seems to give the most reliable result [22, 26–28]. For maximum objectivity our protocol was performed with wide velocity spectrum (90°/s – 360°/s). The 90°/s speed with 3 repetitions was chosen to assess maximum strenght. While 270°/s speed with 15 repetions was used to evaluate the endurance.

We found that peak moment was always lower in external rotation in operated shoulder in comparison to non-operated limb; in two velocities (270°/s in women group and 180°/ in men group) these differences were significant. In internal rotation the relation was opposite. The most comparable to our results were results reported by Amako et al.; however, they were assessing shoulder muscle biomechanical parameters after open Bankart–Bristow method. With a similar test methodology, 12 months after surgery, they achieved results of 23 Nm for external rotation in the operated limb and 24 in the sound limb and for internal rotation: 41 and 46 Nm, respectively [6]. The same authors have also examined patients, male only, over a 5-year period and obtained a result of 24 Nm for ER and 38 Nm for IR [2]. The slightly higher results in comparison to ours may be explained by the higher speed used in the present study. In a group of patients 24 months after arthroscopic labral repair, the PMs were 41.8 and 12.2 Nm in ER and IR, respectively [8].

Amako el al. analyzed patients after arthroscopic shoulder stabilization and found lower TW values in ER after surgery in treated shoulder in comparison to contralateral side. However, they were not significant [2]. Our results considering postoperative TW in ER are similar with Amako et al. [2].

The ER/IR ratio is a parameter which shows the muscle equilibrium during shoulder rotation movements and is very important in terms of the risk of re-injury of the shoulder. Based on studies by Davies, Dvir and Perrin, norms of the shoulder muscle balance of the internal and external rotators group range from 64 to 71% (0.64–0.71) [21–23]. Their calculations were based on peak moment and present ER to IR strength dependency. Brown states that 90°/s and 270°/s velocities seems to be most appropriate for muscle balance examination [29]. In set with the lowest velocity (90°/s) the noticeable resistant is the highest. The trail with 270°/s velocity is the one with the biggest number of repetitions. In women group the ratio parameter was lower than reference values in operated shoulder (Table 4). In men group the ratio parameter was corresponding with reference values. The reason for it is not obvious. Literature states that that women are more muscle fatigue resistant than men [30, 31]; however, this assumption requires further research. Comparable relation was stated by Amako et al. In their research agon/antagon ratio was lower than reference values as well [2]. Tahta et al. found opposite relationship—internal rotation strength was lower than external rotation strength which results in a several hundred percent values of ratio [8]. It could be an argument for taking under consideration the importance of proper muscle balance during rehabilitation program.

For clinical evaluation we used ASES and UCLA scores. Differences between pre and post-surgery results were statistically significant. ASES score results before surgery correlated with PM parameters. In women group patients with better function in clinical assessment before surgery achieved higher values of PM parameters after operation. In men group this dependence was inverse; however, only in women group correlation was moderate. UCLA score correlated with pain before and after surgery in both groups significantly. Patients with less pain achieved better functional results.

### Study limitations

We are aware that the prospective type of this study with biomechanical examination would provide more accurate data. In our study, functional evaluation is prospective but due to high risk of shoulder dislocation the isokinetic examination was performed only after surgery when proper shoulder stability was regained.

## Conclusions

Clinical results measured with ASES and UCLA scores show significant improvement of limb function and reduction of pain and instability.

The values of peak moment and muscle power parameters were slightly lower in external rotation in operated shoulder in comparison to non-operated shoulder. Total work parameter in operated shoulder was significantly lower after surgery in comparison to healthy side. It is related to compromised balance of rotator muscles group and, in our opinion, should serve as a recommendation to focus more attention on muscle balance during rehabilitation program.
