# Correction to: Isokinetic and functional shoulder outcomes after arthroscopic capsulolabral stabilization

**DOI:** 10.1007/s00402-022-04352-1

**Published:** 2022-02-01

**Authors:** Breborowicz Ewa, Lubiatowski Przemyslaw, Jokiel Marta, Breborowicz Maciej, Stefaniak Jakub, Zygmunt Adam, Wojtaszek Marcin, Kaczmarek Piotr, Romanowski Leszek

**Affiliations:** 1 Orthopaedics, Traumatology and Hand Surgery Department, Poznan University of Medical Sciences,, 28 Czerwca 1956 no 135/147, 61-545 Poznań, Poland; 2 Rehasport Clinic, Gorecka 30, 60-201 Poznań, Poland; 3 Physiotherapy Department, Poznan University of Medical Sciences, 28 Czerwca 1956 no 135/147, 61-545 Poznań, Poland

## Correction to: Archives of Orthopaedic and Trauma Surgery 10.1007/s00402-021-04290-4

The original version of this article unfortunately contained a mistake. Two rows are missing in the Table 2.

The corrected Table 2 is given in the next page.

**Table 2:** Values of peak moment (PM), peak moment/body weight (PM/BW), average power (AVG POWER), total work (TW) and average peak moment (AVG PM) parameters in external and internal rotation with comparison between operated and non-operated side in women group

Sex	Trial	External rotation										Internal rotation									
		PM		PM/BW		AVG POWER		TW		AVG PM		PM		PM/BW		AVG POWER		TW		AVG PM	
		nop	op	nop	op	nop	op	nop	op	nop	op	nop	op	nop	op	nop	op	nop	op	nop	op
WOMEN	90°∕s																				
	Avg	15.2	13.3	22.3	19.6	11.7	9.4	44.7	34.3	13.9	12.2	23.4	22.3	34.3	32.8	18.5	17.9	72.9	67.8	20.5	21
	SD	3.6	3.2	7.1	5.2	3.5	3.9	13.6	15.2	3.6	2.7	3.8	7.5	6.6	11.5	5.3	8.7	21.3	34.1	3.8	6.9
	p	NS		NS		NS		0.02*		NS		NS		NS		NS		NS		NS	
	180°∕s																				
	Avg	16	13.9	23.5	20.6	7.5	8.7	24	23.1	13.6	12	16.7	20.2	25	30	8.6	13.9	26.9	37.7	14	17.7
	SD	5.0	4.3	8.1	7.1	3.5	4.9	8.9	10.4	4.4	4.1	6.9	6.4	11.8	9.3	5.2	11.6	15.3	27.0	5.8	6.2
	p	NS		NS		NS		NS		NS		NS		NS		0.03**		0.04**		0.04**	
	270°∕s																				
	Avg	19.2	15.7	28.4	23.3	13.0	11.2	161.5	126.5	16.1	13.1	27.9	28.7	41.7	42.7	21.0	17.8	248.3	225.2	23.8	24.4
	SD	4.3	5.1	7.9	7.9	6.3	5.0	47.9	50.7	3.8	4.5	6.7	8.1	12.2	13.4	10.1	11.7	96.4	117.1	6.4	7.6
	p	0.04*		NS		NS		0.04*		NS		NS		NS		NS		NS		NS	
	360°∕s																				
	Avg	15.6	13.1	23	19.5	13.5	10.8	31.8	25.3	14.4	11.8	24.0	23.3	35.4	34.5	18.8	18.2	48.1	45.0	21.6	21.4
	SD	4.8	3.8	8.3	6.5	6.7	6.0	12.1	10.8	4.8	3.5	8.9	9.0	14.1	13.7	9.5	12.6	19.3	22.2	8.3	8.9
	p	NS		NS		NS		0.04*		NS		NS		NS		NS		NS		NS	

*t-Student test

**Wilcoxon test

The original article has been corrected.

