# Correction to: Isokinetic and functional shoulder outcomes after arthroscopic capsulolabral stabilization

**DOI:** 10.1007/s00402-023-05043-1

**Published:** 2023-09-11

**Authors:** Ewa Breborowicz, Przemyslaw Lubiatowski, Marta Jokiel, Maciej Breborowicz, Jakub Stefaniak, Adam Zygmunt, Marcin Wojtaszek, Piotr Kaczmarek, Leszek Romanowski

**Affiliations:** 1 Orthopaedics, Traumatology and Hand Surgery Department, Poznan University of Medical Sciences, 28 Czerwca 1956 no 135/147, 61-545 Poznań, Poland; 2 Rehasport Clinic, Gorecka 30, 60-201 Poznań, Poland; 3 Physiotherapy Department, Poznan University of Medical Sciences, 28 Czerwca 1956 no 135/147, 61-545 Poznań, Poland

**Correction to: Archives of Orthopaedic and Trauma Surgery (2022) 142:3927–3935** 10.1007/s00402-021-04290-4

The original version of this article unfortunately contained a mistake. The given name and family names were interchanged.

The correct given name and family names should be:

Ewa Breborowicz

Przemyslaw Lubiatowski

Marta Jokiel

Maciej Breborowicz

Jakub Stefaniak

Adam Zygmunt

Marcin Wojtaszek

Piotr Kaczmarek

Leszek Romanowski

The original article has been corrected.

